# Inclusion Complexes of Concentrated Orange Oils and β-Cyclodextrin: Physicochemical and Biological Characterizations

**DOI:** 10.3390/molecules25215109

**Published:** 2020-11-03

**Authors:** Cynthia Torres-Alvarez, Sandra Castillo, Eduardo Sánchez-García, Carlos Aguilera González, Sergio Arturo Galindo-Rodríguez, José A. Gabaldón-Hernández, Juan G. Báez-González

**Affiliations:** 1 Facultad de Ciencias Biológicas, Universidad Autónoma de Nuevo León (UANL), Av. Pedro de Alba s/n, Cd. Universitaria, C.P. 66455 San Nicolás de los Garza, Nuevo León, Mexico; cta_83@hotmail.com (C.T.-A.); sandra.castilloh@uanl.mx (S.C.); eduardo.sanchezgrc@uanl.edu.mx (E.S.-G.); carlos.aguileragn@uanl.edu.mx (C.A.G.); sagrod@yahoo.com.mx (S.A.G.-R.); 2 Departamento de Ciencia y Tecnología de Alimentos, Universidad Católica San Antonio de Murcia (UCAM), Avenida de los Jerónimos s/n, Guadalupe, 30107 Murcia, Spain; jagabaldon@ucam.edu

**Keywords:** β-cyclodextrin, inclusion complex, fold orange essential oil

## Abstract

Concentrated orange oils (5x, 10x, 20x) are ingredients used in different industries as components of flavors and aromas due to their great organoleptic qualities. This research focuses on the search for alternative uses for their application through encapsulation in inclusion complexes with β-cyclodextrin (β–CD). Inclusion complexes of concentrated orange oils (COEO) and β–CD were developed by the co-precipitated method in ratios of 4:96, 12:88, and 16:84 (*w/w*, COEO: β–CD). The best powder recovery was in the ratio 16:84 for the three oils, with values between 82% and 84.8%. The 20x oil in relation 12:88 showed the highest entrapment efficiency (89.5%) with 102.3 mg/g of β–CD. The FT-IR analysis may suggest an interaction between the oil and the β–CD. The best antioxidant activity was observed in the ratio 12:88 for the three oils. The antifungal activity was determined for all the inclusion complexes, and the 10x fraction showed the highest inhibition at a concentration of 10 mg/mL in ratios 12:88 and 16:84. Antibacterial activity was determined by the minimum inhibitory concentration (MIC) and was found at a concentration of 1.25 mg/mL in ratios 12:88 and 16:84 for 5x and 20x oils.

## 1. Introduction

Plants produce secondary metabolites as essential oils, being a complex mixture of volatile organic compounds as hydrocarbons (mono- and sesquiterpenes) and oxygenated compounds (alcohols, esters, ethers, aldehydes, ketones, among others) [1]. From a few years ago until now, the scientific interest in essential oils (EOs) and extracts has increased due to their diverse applications in different markets; this is because they have a wide range of uses in the food industry (e.g., antifungal and antioxidant agents, flavoring), in the cosmetic industry, in the cleaning industry (due to their aromatic properties), in the pharmaceutical industry (including preventive properties against cancer, diabetes, inflammations and antiviral uses), as well as in other industries such as the chemical and agricultural industries; also, essential oils are considered (generally recognized as safe (GRAS) by the US Food and Drug Administration (FDA) [2,3,4].

**Figure 1:**
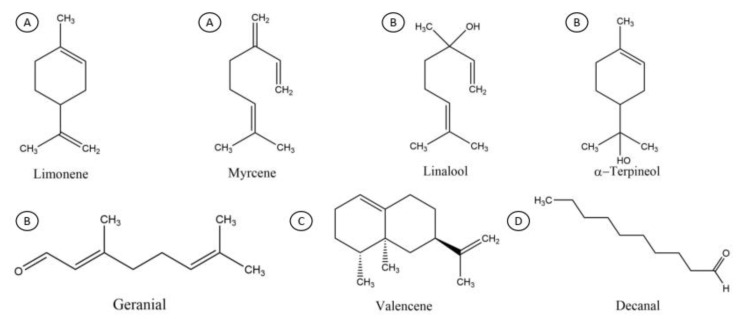
Chemical structures of representative major compounds for the orange essential oil and its concentrated oils, ordered as: (**A**) hydrocarbon monoterpenes; (**B**) oxygenated monoterpenes; (**C**) hydrocarbon sesquiterpenes; (**D**) oxygenated compounds.

Cold pressing is the method for obtaining citrus essential oils directly, wherein oil glands (the external part of the mesocarp) are pressed so they release an essential oil, which is then centrifuged to remove impurities; this is called single-fold oil (1x) [3,5]. The orange essential oil consists mainly of oxygenated, terpenes, and sesquiterpenes as representative major compounds: Limonene, Myrcene, Linalool, α-Terpineol, Decanal, Geranial and Valencene (Figure 1); these latter compounds are considered chemically unstable and easily oxidized in the presence of air, light, and moisture [6]. Conventionally, high vacuum fractional distillation is used for obtaining fold oils (concentrated) from citrus essential oils; this process aims at the separation of two or more substances by the volatility difference between them. The pressure and temperature of the system, as well as the physical and chemical characteristics of the components to be separated are important factors to consider [7]. The monoterpenes, mainly limonene, (representing around the 80% of the composition for the citrus essential oils) are partly removed (depending in the folding degree) through this process due to several disadvantages such as their instability in heat and light and their insolubility in water; also, they do not contribute much in flavor. When such types of compounds are removed, other fractions with more industrial interest are enriched, e.g., the oxygenated compounds, which provide much of the characteristic flavor of citrus oil [8,9], deriving a new concentrated product called folded oil e.g., five-fold concentrate, which is concentrated to 1/5 of its original weight [9,10]. Commercially, the main folding degrees available are: 2-fold (2x) to 5-fold (5x) for mandarin and lime oil; 2-fold (2x) to 10-fold (10x) for lemon and grapefruit oils; and 2-fold (2x) to 20-fold (20x) for orange oil known as concentrated orange essential oil (COEO). With the folding process, the citrus oils increase their amount of oxygenated (e.g., decanal, octanal, linalool, citral) and sesquiterpenes compounds (e.g., valencene, nootkatone) [10,11].

In the food industry, there is a great interest in plant extracts, such as essential oils, due to their antioxidant and antimicrobial activities, as natural food preservatives, and because of their flavor retention capacity; however, their use is limited because of their volatility and low solubility in water [6]. Moreover, essential oils are applied as a non-synthetic antimicrobial source for edible films for controlling the growth of microorganisms in real-food samples [12]. For these reasons, essential oils require the protection provided by microencapsulation. The use of cyclodextrins represents one of the simplest yet most efficient encapsulation systems [13].

Cyclodextrins (CDs) are cyclic oligosaccharides consisting of α–d-glucopyranose units attached to (α-1,4) in a ring formation. The CDs are derived from the enzymatic digestion of starch by the enzyme cyclodextrin glycosyltransferase, forming a hydrophilic exterior and an internal hydrophobic cavity [14,15,16]. The most common native CDs are α-cyclodextrin (α-CD), β–cyclodextrin (β–CD), and γ–cyclodextrin γ–CD) formed by six, seven or eight glucose units, respectively. The cavity diameter is determined by the number of glucoses, which in turn controls the size of the guest molecule that can be included into the cavity [15,17]. Among the cyclodextrins, the β–CD has been widely applied due to its availability, suitable cavity size, ease of production, and for the high thermal stability that it confers on encapsulated compounds [6].

CDs are mainly used in the formation of inclusion complexes, which has allowed their use for specific purposes in the food, cosmetic, pharmaceutical, and textile industries [14]. For example, in the pharmaceutical area, studies have been carried out on increasing the bioavailability of poorly soluble drugs [18], the use of materials such as nanoesponges, as well as β-cyclodextrin for the administration of multiple drugs [19], in addition to structures such as self-assembled cyclodextrin nanomaterials [20]. In the food area, CDs have been widely used as food additives, to stabilize sensitive ingredients to light and temperature (e.g., flavors, natural compounds as essential oils), to eliminate undesired tastes, to prevent oxidation during storage, to improve flavor retention in thermally processed food, to improve the color and/or aroma of different fruit juice, and to create a controlled release system of active substances [6,15,21,22]. Industrially, inclusion complex formation by cyclodextrins has been used to increase the solubility of functional ingredients [23] and to improve the sensorial properties of food [21]. The encapsulation performance with β–CD of some oils such as garlic [22], orange, eucalyptus [6], thyme [24], and pure compounds such as limonene [25], linalool [26], citronella, citronellal, citronellol [27], estragole [15], trans-anethole [13], eugenol, carvacrol [12], polyphenols (such as chlorogenic acid) [28], and mangiferin [29] have demonstrated an effective interaction.

Encapsulation and its effect on the physicochemical properties and the bioactivity of concentrated orange oils (COEO) have not been reported in any research. The aim of the present study was to produce the inclusion complexes of the concentrated orange oils (5x, 10x, and 20x) with β–CD in various ratios (COEO:β–CD) 4:96, 12:88, and 16:84 using a co-precipitation method to evaluate the recovery (%), entrapment efficiency (%), morphology and particle size (μm), interaction (FT-IR), and biological activity.

## 2. Results and Discussion

### 2.1. Characterization of COEO Inclusion Complex

#### 2.1.1. Recovery of COEO Inclusion Complex

The percentage of powder recovery was calculated by the difference between the amount of β–CD and the essential oil originally used and the final weight of the recovered powder. The recovery of the powder at equilibrium state for each COEO at β–CD ratios are presented in Table 1. The statistical comparison of data shows significant differences between treatments with values ranging from 74.4% to 84.8%. The lowest recovery was found in the 4:96 ratio for all oils. The maximum inclusion in β–CD was achieved at the 16:84 and 12:88 ratios with values above 80%. These results are similar to those of some authors who have performed complexes with cinnamon oil, garlic, thymol, and *Satureja montana* with similar recovery results [24,30,31,32]. However, there are some factors that may contribute to the loss of COEO: retention of the oil in the solution after forming the complex, equilibrium of flavors between the liquid and the complexed state, evaporation of surface oil during the long complexation process, or evaporation of surface oil during the drying step. On the other hand, the loss of the β–CD and complex powder is mainly related to the solubility of β–CD in water [31].

#### 2.1.2. Entrapment Efficiency (%EE)

The entrapment efficiencies of COEO: β–CD inclusion complexes prepared by co-precipitation method are shown in Table 2. The %EE values ranged from 67.1% to 89.5%. The values above 80% were found in ratios of 4:96 for 10x and 20x, 12:88 for all oils, and 16:84 only for 5x. Results are consistent with other previous studies [15,33,34], which had studied different compounds such as eugenol and carvacrol, among others. They reported %EE values between 17% and 99%. In this investigation, the results show an efficiency greater than 70%, with values around 80%. Entrapment efficiency varied depending on the COEO used and the performed ratio, 12:88 ratio being the one that showed a high and stable entrapment efficiency in all cases. Dos Passos et al. [25] reported low values of complexation efficiency of the limonene inclusion complexes, possibly because limonene remained on the surface of the complex so it was not fully integrated; also, because of other factors involved in the process as volatilization by the heating and drying steps. Another factor to consider is the chemical profile of each COEO (5x,10x, 20x) (data not shown) that could modify the %EE; in a previous study performed in our laboratory, we reported the composition of these concentrated oils that showed a decrease in volatile compounds such as limonene in ranges of 28% to 80% compared with the orange essential oil. This led to the increase in other compounds with higher molecular weight [11]. These compounds may be more difficult to complex due to the size of the β–CD cavity than volatile compounds with lower molecular weight, since they may compete for inclusion reactions with the β–CD; when the volatile compound is not present, there is no competition, and the remaining compounds are easier to complex. Different molecules have different equilibriums in a solution, and that drives them either to form inclusion complexes or to remain in solution [35]. On the other hand, the method selected may be crucial in the EE percentage. Wang et al. [22] reported EE of 90.3% when they encapsulated garlic through the co-precipitation method, while in this same method, Zhang [13], reported 51.29% using Trans-anethole. Santos et al. [33] reported a 91.3 % EE of complex carvacrol:β–CD by freeze-drying at low temperatures but 83.8% EE by kneading. Considering all the data mentioned above, the EE values obtained in this study for co-precipitation method are in acceptable ranges (% EE: 67.1–89.5).

The encapsulated oil values ranged from 7.7 to 102.3 mg/g β–CD; they are shown in Table 2. The quantity of encapsulated oil varied between COEO (5x, 10x, 20x) and respective ratios (4:96, 12:88, 16:84). The 20x COEO showed the greater encapsulation capacity in all ratios with significant differences between the other COEO (5x, 10x) and their respective ratios. For the rest of the oils and ratios, there were significant differences when obtaining values < 60 mg/g β–CD. In general, the ratio of 4:96 had the lowest interaction between COEO and β–CD due to the low values of encapsulated oil, while 12:88 seems to have the greatest interaction with exception of the 5x oil. Madene [36] reported that the inclusion complexes are the result of interactions between compounds, which allow the smaller molecules to fit more easily inside the cavity of the cyclodextrin. Petrović et al. [31] reported that an optimum ratio for inclusion complexes with cinnamon oil was 15:85, obtaining 117.2 mg/g β–CD; he mentions that the polarity of the compounds could play an important role in the complexation, being that less polar compounds are easier to complex than more polar compounds. These results may suggest that the maximum inclusion of β–CD with 20x COEO could be attributed to the low polarity of the oil or the kind and quantity of compounds present. 

#### 2.1.3. Particle Size and Morphology

The average particle sizes are shown in Table 3. The average particle sizes were significantly higher in the ratio of 4:96 for all oils, showing significant differences compared with the 12:88 and 16:84 ratios. These results are consistent with those reported by Tao et al. [35] with thyme oil (3.2 μm), who states that the method used to perform the inclusion complexes may influence the particle size through the complexation time during the agitation process. However, β–CD has a strong tendency for agglomeration as a consequence of its self-assembly in water [37] due to the lack of significant net charge on the inclusion complex particles; this means there are no repulsive forces to prevent particle agglomeration [38]. Cyclodextrins and their complexes may form water-soluble aggregates in aqueous solutions and these aggregates may be capable to solubilize lipophilic drugs by non-inclusion complexation or micelles-like structures [35].

**Figure 2:**
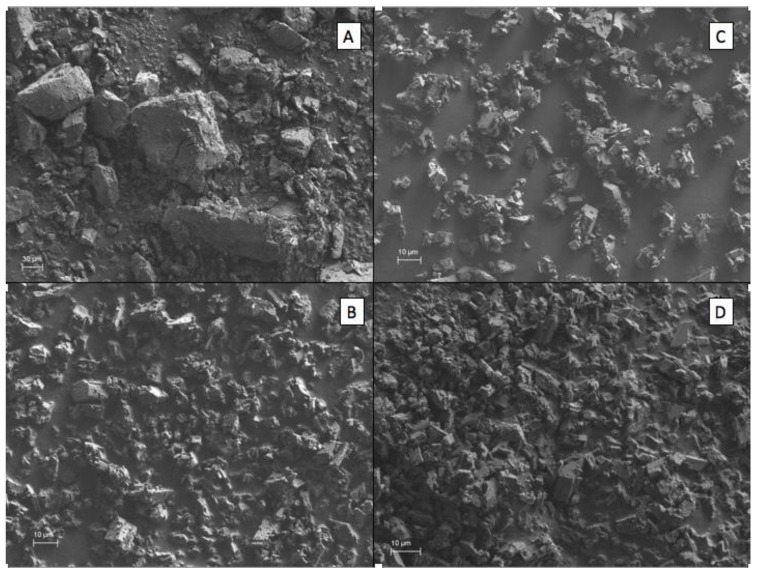
Surface morphologies of the 12:88 ratio compared with pure β–CD as a control. (**A**) Pure β–CD, (**B**) 5x-β–CD, (**C**) 10x-β–CD, (**D**) 20x-β–CD.

The surface morphologies of the β–CD, as well as the inclusion complexes, are shown in Figure 2. The morphology of the inclusion complexes was analyzed through scanning using an electron microscope (SEM) in order to obtain the images and to observe if there is a morphological difference between pure β–CD and the inclusion complexes. In this study, the 12:88 ratio was the most used since it was the one that presented the best results in most of the analyses performed. The morphology shows a compact particle of amorphous and irregular shape with tendency towards a rectangular shape at different sizes in the pure β–CD. Zhang et al. [13] analyzed the surface morphology of the complexes made with trans-anethole (aromatic compound of the anise) obtained through the co-precipitated method and the physical mixture, observing that it had amorphous and irregular crystalline structures and physical changes depending on the technique used. They concluded that morphologies were similar to the β–CD, but smaller in size or altered in shape. Some authors mention that the alteration or change of crystal structures and dust suggests the formation of inclusion complexes with cyclodextrin [28,39,40]. In our study, we obtained differences in shape and size compared with the control; these findings could suggest the formation of inclusion complex of concentrated oils and β–CD by using the co-precipitation technique.

#### 2.1.4. FT-IR Analysis

**Figure 3:**
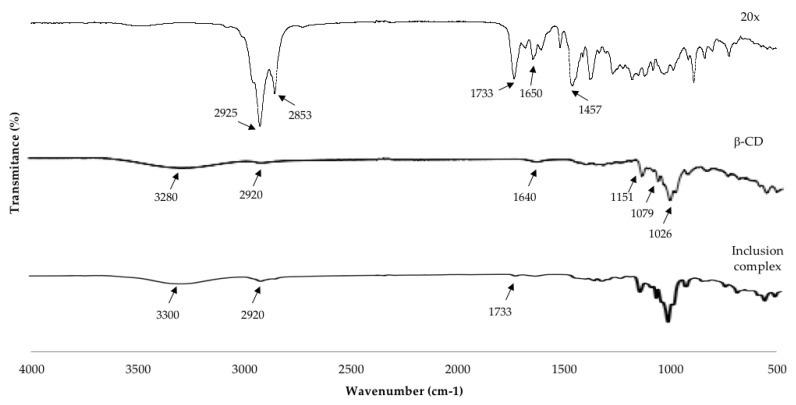
FTIR spectra of 20x, β–CD, inclusion complex (20x-β–CD).

The FTIR analysis is a widely used technique to characterize β–CD inclusion complexes and mainly analyzes the changes in the spectra of the guest molecules by the molecular interaction between β–CD and oils [12]. The FTIR spectra of the essential oil (20x), the pure β–CD, and inclusion complexes (20x: β–CD) are presented in Figure 3. The FTIR spectrum of the pure β–CD showed a broad band with a maximum absorption centered at 3280 cm^−1^ corresponding to the O-H stretching vibration of the different hydroxyl groups. The spectrum presented another band, mainly at 2920 cm^−1^ (C-H stretching vibration of CH and CH_2_ groups), 1733 cm^−1^ (C=O stretch), a slight band also visible at 1640 cm^−1^ related to the bending vibration of H-O-H, at 1455, 1412, 1347 cm^−1^ due to C-H bending vibrations. Absorption bands at 1151, 1079, and 1026 cm^−1^ are attributed to the C-O stretching vibrations. Finally, the rocking vibration of the C-H bonds and the C-C skeletal vibrations in the glucopyranose ring correspond to bands in the region 940–700 cm^−1^ (not all the bands are marked). Menezes et al. [26] obtained similar results investigating inclusion complexes of β–CD with linalool. The FTIR spectrum of the concentrated oil (20x) in the 2925–2853 cm^−1^ range showed some bands that correspond to different C-H bonds that could be expected in 20x. Furthermore, stretching vibrations (ν) in the bands of the group C=C in the range between 1650–1450 cm^−1^; in the range of 3000–2800 cm^−1^ they correspond to C-H stretching vibration [1]. Furthermore, the band in 1457 cm^−1^ may be associated with C=C elongation vibrations of the oleophilic groups of essential oils. The spectra of inclusion complexes 20x/β–CD were observed the typical bands by β–CD, showing at 3308 cm^−1^ (for O-H stretching vibration) and a considerable decrease in intensity in the absorption band by 2920 cm^−1^; the reduction of characteristic peaks in 1733–1183 cm^−1^ in 20x, providing information about the possible interaction of the 20x in the cavity of β–CD, therefore it could suggest the formation of oil–β–CD inclusion complexes. The guest molecule could have some changes in its characteristic bands such as disappearance, broadening, and variations in the peak intensity indicate the restriction of the stretching vibration because the insertion of the guest molecule was included into the β–CD cavity, which consequently reduced the movement of the encapsulated molecules [6]. Similar studies which have evaluated the inclusion complexes with thyme oil, limonene, eucalyptus, and orange oil observed the disappearance of some bands or a reduction of the intensity of some bands characteristic of essential oils; they suggest the interaction of essential oil inside the β–CD cavity [1,6,24,25]. Each inclusion complex (oil:β–CD) of 5x and 10x showed the same tendency (data not shown).

### 2.2. Biological Assay

#### 2.2.1. Antioxidant Activity

**Figure 4:**
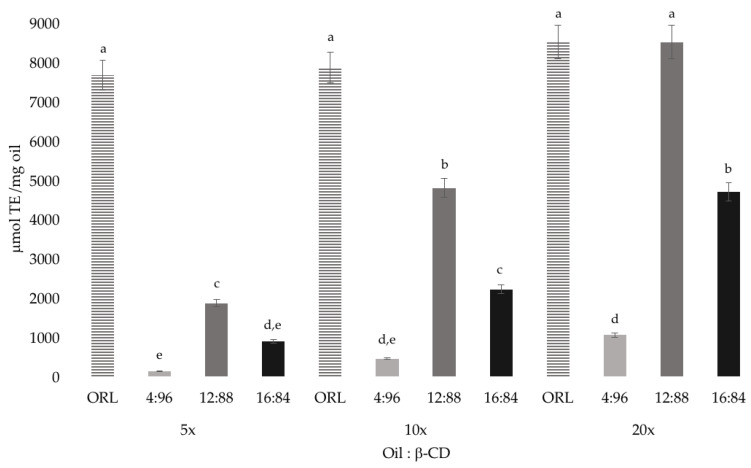
Antioxidant activity of the inclusion complexes of concentrated orange oils with β–CD in different ratios. Data were analyzed by an analysis of variance test (one-way ANOVA) and a post hoc test of Tukey’s multiple range. Different letters (a, b, c, d, e) above bars are significantly different (*p* < 0.05).

The results obtained regarding the antioxidant activity of concentrated oils 5x, 10x, 20x free (ORL, bulk oil) and inclusion complex (IC) in their different ratios with the β–CD (Figure 4), showed values between 137–8500 μmol of Trolox/mg oil. Results showed no significant difference between concentrated oils without encapsulating and inclusion complexes in the 12:88 ratio for the 3 oils with values between 1865–8500 μmol Trolox/mg oil. However, a significant difference was evidenced between different ratios being 12:88 the one that presented greater activity; however, it was lower for the 4:96 and 16:84 ratios. The oil 5x showed the lowest activity in relation 4:96 and 16:84 with values ranging between 137–2000 μmol of Trolox/ mg oil. No decrease in the absorbance of the DPPH and 2,2′-azinobis-3 ethylbenzthiazoline-6-sulphonate (ABTS) solutions was observed in the presence of β–CD alone, therefore, the β–CDs have no activity for the inhibition of these radicals. Numerous studies have investigated whether the active compound continues its biological action even when it is inside the β–CD cavity. In antioxidant activity, free radicals such as DPPH and ABTS are widely used for the study of natural compounds [40]. Several researchers have encapsulated cyclodextrins using different techniques; it has been done with some pure compounds such as resveratrol, estragole, chlorogenic acid, mangiferin and essential oils of basil and tarragon, studying whether these compounds maintain the antioxidant activity they possess, even with the presence of cyclodextrins [15,28,29,40].

#### 2.2.2. Antifungal Activity

The antifungal activity test was carried out using *Aspergillus niger* and *Aspergillus flavus* fungi due to their great importance in the food industry and because they are microorganisms that produce mycotoxins. Diminution in mycelial growth was measured with a symbolic-arbitrary scale (representing with +), while Fungal Inhibitory Concentration (FIC) was defined as the minimum concentration in which there was no visible mycelial growth. β–CD was used as negative control and did not cause a reduction in mycelial growth. Antifungal activity was determined in not encapsulated concentrated oils 5x, 10x, 20x and in its three relations with the β–CD (4:96, 12:88, 16:84), using the concentrations of 1.25, 2.5, 5 and 10 mg/mL. The results showed a diminution in mycelial growth in a concentration-dependent manner only for the non-encapsulated COEO (Table 4), in which FIC was determined with this method at 10mg/mL for the 5x, 10x and 20x with exception of *A. flavus* in which the 10x fraction showed a diminution but not absence of mycelial growth. The FIC could not be determined for the COEO:β–CD because there was no complete inhibition in none of the concentrations tested, and the ratio 4:96 did not show activity for any of the oils tested. However, *A. niger* was more susceptible than *A. flavus* in all cases and the encapsulated 10x oil (12:88, 16:84) showed major inhibition in a concentration of 10 mg/mL. The encapsulated 5x oil showed a slight inhibition in the concentration of 10 mg/mL in all cases. On the other hand, with the exception of the *A. flavus* (β–CD 16:84, 10 mg/mL), the encapsulated 20x oil did not show inhibition for any of the concentrations tested. Although the 20x β–CD complex contains more oil, it does not necessarily have to be more active, because the compounds found in this concentrated oil are different from the other two. The 20x oil contains very low amounts of limonene compared to the 10x fraction [11], it also contains other compounds in greater quantities that can compete with limonene and enter in the cavity of the complex [31]. This can cause a decrease in antimicrobial activity because the compounds complexed may have less activity. It is known that the antimicrobial activity is due to the synergy between several compounds [35], synergy that could be lost by missing key compounds in the antimicrobial activity during the complexation. Haloci et al. [32] investigated the inhibition of *Satureja montana* oil using the ratios 5:95, 10:90, and 20:80 with eight species of fungi, reporting areas of inhibition from 3.13% to 100% in inclusion complexes, depending on the relationship of the fungus and the strain being studied; the most effective relationship for antifungal activity was 10:90. Kfoury et al. [15] studied the antifungal activity in some phenylpropanoids such as stagol, eugenol, among others, free-form and encapsulated, against two fungi of importance in post-harvest as *Fusarium oxysporum* and *Botrytis cinerea*; this author reported that the encapsulation with cyclodextrins increased the solubility and photostability of the compounds studied and their remarkable antifungal properties, showing low values of inhibitory concentration in the growth of the mycelium and germination of the spores. However, the results of this investigation did not show a high inhibition at low concentrations of the complexes compared with not encapsulated COEO, and this may be due to the concentration of the oil within of the complexes of β–CD which may not have been enough to cause the complete inhibition of the fungus. Our results are consistent with those reported by Del Toro et al. [24], in which β–CD complexes of thyme oil caused only a diminution in the fungus growth, and thus were less effective than free oil. Ayala-Zavala et al. [30] report mycelial growth of *Alternaria alternata* treated with cinnamon oil and garlic in the ratios 12:88 and 16:84 in different concentrations of inclusion complex, mentioning that responses depended on the type of fungus, the requirements for its growth, and the inhibitory capacity that the component or components in the study could have. 

#### 2.2.3. Antibacterial Activity

**Figure 5:**
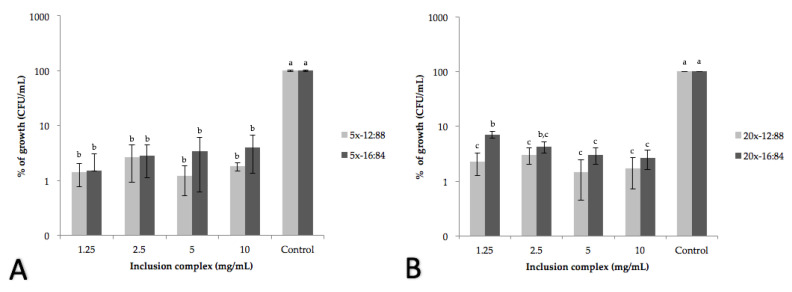
Growth of *Salmonella typhi* (**A**) and *Listeria monocytogenes* (**B**) at different concentrations of 5x and 20x concentrated oil inclusion complexes at 12:88 and 16:84 ratios, respectively. Data were analyzed by analyses of variance test (one-way ANOVA) and a post hoc test of Tukey’s multiple range. Different letters (a, b, c) above bars are significantly different (*p* < 0.05).

The antibacterial activity test of the inclusion complexes was carried out evaluating the minimum inhibitory concentration (MIC) of each COEO: β–CD against important strains in the food industry such as *Salmonella typhi* and *Listeria monocytogenes.* In previous studies in our laboratory, we reported the MICs of the non-encapsulated concentrated oils the 5x and 20x fractions being the ones that showed the strongest antimicrobial effect for *Salmonella typhi* and *Listeria monocytogenes,* respectively [11]. When performing the MIC analysis with COEO: β–CD, we observed that the bacterial growth decreased more than 90% in both oils, obtaining less than 10% of growth in all cases for both strains (Figure 5). The concentration of inclusion complexes required to inhibit microbial growth at 90% is 1.25 mg/mL for both bacteria in the 12:88 and 16:84 ratios, with no significant differences between the concentrations tested, but compared to the control. None of the COEO: β–CD caused a complete inhibition and there was not concentration dependent behavior between the concentrations tested but a bacteriostatic effect was observed. According to the results presented by Torres-Alvarez et al. [11], in which MIC and minimum bactericidal concentration (MBC) were determined for the not encapsulated COEO (ranging from 0.25 to 2.5 mg/mL) this last was more effective against bacteria than the encapsulated COEO. These results are consistent with Duarte et al. [40] who report major antimicrobial activity of free resveratrol than those encapsulated with β–CD. This could be attributed to the stability of the β–CD-complexes and the low dissociation rate of the oil in the media that provoke a lower contact of the antimicrobial compounds with the bacteria. This situation also could cause the bacteriostatic effect, because the concentration of the oil in the media is not enough to cause a concentration dependent diminution or total inhibition in bacterial population. In this respect, Mayaud et al. [41], reported bacteriostatic effect of different concentration (0.04, 0.08, 0.016%) of cinnamon bark essential oil concluding that the presence of certain predominant chemical groups determines the bacteriostatic activity of an essential oil while the bactericidal effect is achieved at higher concentrations. The importance of encapsulating a compound is that even if it is coated, it can be released and still preserves the activity it presents in its free form; therefore, the food industry can consider natural compounds to exert inhibitory action on bacteria. In general, according to the results obtained in this study, the inclusion complexes had antimicrobial properties evidenced by the inhibition of bacterial growth. Numerous investigations have studied the application of cyclodextrins, generating inclusion complexes with essential oil compounds of cinnamon, clove, thyme as trans-cinnamaldehyde, eugenol, thymol, carvacrol, resveratrol against bacteria such as *Escherichia coli*, *Salmonella typhimurium*, *Listeria innocua*, and of the genus *Campylobacter* [33,35,38,40], improving in some cases the antimicrobial efficiency of the oil at a lower concentration; however, that depends a lot on the type of compound and the strain, because some compounds are more efficient for some strains. Hill et al. [38] reported lower MIC to inhibit the growth of *Salmonella typhimurium* and *Listeria innocua* in compounds such as trans-cinnamaldehyde, eugenol, cinnamon extract, and clove, requiring for their inhibition of 4.2% to 86% less of the compound using 0.16 to 1.15 mg/mL of inclusion complexes; this may depend on the resistance of the strain to the component, due to the fact that β–CD manages to improve the antimicrobial capacities of essential oils, since the antimicrobial action of these occurs in different ways, some in the membrane and inside the cytoplasm of bacteria. It is also possible that the technique used to obtain the inclusion complexes may have an influence. Tao et al. [35] reported for the thymol and thyme oil component, using the methods of obtaining kneading and cold drying, that the latter was more efficient for the preparation of the complexes because it achieved a greater inhibition of *Escherichia coli* K12 with lower concentrations of both compounds in comparison to the kneading technique; however, another investigation in which the same techniques were used for the preparation of inclusion complexes but with carvacrol, the kneading technique was more effective to inhibit the growth of *Escherichia coli* K12 and *Salmonella typhimurium,* obtaining inhibitions close to 70%.

## 3. Materials and Methods

### 3.1. Materials

#### Concentrated Orange Oils

Concentrated orange oils (COEO) (5x, 10x, and 20x) were provided by Frutech International (Guadalupe, Nuevo Leon, Mexico), and were industrially obtained through the cold-pressing method; subsequently, the folded oils were prepared in a vacuum distilled column with structured packing equivalent to 20 theoretical plates. The equipment was operated in a batch mode. The conditions of the parameters used during vacuum distillation were adjusted based on the initial composition of orange essential oil in order to obtain a final product with a desired composition, according to the specification of each folded oil with the following parameters: pressure range 5–20 mbar and reflux at temperatures between 80 °C and 100 °C; The physicochemical properties and composition (GC-MS analyses) of each oil was previously reported [11]. β-cyclodextrin (β–CD) was provided by Wacker Chemie AG and Trolox was purchased from Sigma–Aldrich (Sigma Chemical Co., St. Louis, MO, USA). The reagents used were analytical grade.

### 3.2. Formation and Characterization of the Inclusion Complex

#### 3.2.1. Preparation of β-Cyclodextrin Inclusion Complexes 

The precipitation method was used to prepare the oils–β–cyclodextrin complex (COEO inclusion complexes) by Ayala-Zavala et al. [30], with some modifications. Five grams of β–CD were dissolved in 50 mL of ethanol and water (1:2 *v/v*), and maintained at 55 °C (±2 °C) on a hot plate (super-nuova multi-place, thermo scientific). A predetermined quantity of each oil was dissolved in ethanol (10% *w/v*) and then slowly added to the warm β–CD solution using continuous stirring. The following three starting ratios of oils onto β–CD were used 4:96, 12:88, and 16:84 *w/w*, (COEO: β–CD). The choice of these proportions was according to others previously reported and also showed good encapsulation performance [24,30]. The mixture was stirred for another 4 h, without heating, while its temperature decreased to 25 °C. The final solution was refrigerated overnight at 4 °C. The cold precipitated material was recovered through vacuum filtration. The precipitate was dried in a convection oven (model HS, Riossa) at 50 °C for 24 h. The powder was then removed from the oven and allowed to air-dry at 25 °C for an additional 24 h in order to reach its moisture equilibrium content and was then weighed. The amount of powder that was recovered (dry basis) was calculated in percentage from the difference between the initial weight of ingredients used and the final weight of powder recovered. The obtained complex was stored in airtight glass containers at room temperature.

#### 3.2.2. Entrapment Efficiency (%EE)

The amount of each oil (5x, 10x, and 20x) entrapped in each inclusion complex was determined according to Santos et al. [33] with some modifications, by using the turbidity method in a spectrophotometer (model Genesys 10S UV-Vis, Thermo Scientific) at 313 nm for all the oils. The encapsulated oil (entrapped in the β–CD cavity) was calculated from the difference between the total oil and the surface oil of inclusion complexes. For the calculation of total oil, the inclusion complexes were weighted (0.1 g) and washed twice in a solution of distilled water (5.35 mL) and hexane (2.66 mL). The mix was vortexed for 2 min; after that, the organic phase containing the volatile compounds was separated and turbidity was measured in the spectrophotometer. The oil adsorbed on the surface of the inclusion complexes was determined according to Petrović et al. [31], with some modifications. Briefly, samples of 0.1 g were washed with hexane (2 mL) and vigorously shaken. After that, the suspension was filtered and measured in the spectrophotometer. In order to obtain the concentration of the total or surface oil, a calibration curve was prepared with oil concentrations ranging from 0.18 to 2.8 mg/mL.

The difference between the total oil and the surface oil, is the amount entrapped on the complexes in the β–CD cavity. The EE was calculated according to the following Equation (1):

(1) $$\%\mathrm{EE}=\frac{total\;oil-surface\;oil}{total\;oil}\times 100$$

The *total oil* was the amount entrapped in the β–CD cavity plus the surface oil and the *surface oil* was the amount adsorbed on the surface of the inclusion complexes.

#### 3.2.3. Particle Size and Morphology

The average particle size for each type of inclusion complex was measured using a Mastersizer 3000 (Malvern Instruments) with a refractive index of 1.333. The particles were weighed (0.07 g) and dispersed in isopropanol as a dispersing liquid [33].

The morphologies of commercial β–CD and the inclusion complex of COEO: β–CD were obtained in a scanning electron microscope (Merlin VP Compact, Zeiss, Germany). The samples were fixed on the assembly buttons and were observed under a microscope [42].

#### 3.2.4. Fourier Transform Infrared Spectroscopy (FT-IR) Analysis

IR spectra of free β–CD, free (5x, 10x, 20x) and inclusion complexes were recorded using an infrared spectrophotometer FTIR (Perkin Elmer). The scanning conditions were: wave number range from 4000 to 400 cm^−1^; resolution 4 cm^−1^; number of scans 16; scan speed 0.2 cm^−1^; detector LiTaO3 [24].

### 3.3. Biological Assay

#### 3.3.1. Antioxidant Activity Assay

In order to determine the antioxidant activity, 0.1 g of each inclusion complex was placed in a centrifuge tube containing 1.5 mL of a solution of NaCl (10%)–methanol (1:1). The sample was then vortexed for 10 min, and 8 mL of a hexane–acetone (1:1) solution was added and centrifuged at 10,000 rpm for 15 min. The antioxidant activity was measured using the 2,2′-azinobis-3 ethylbenzthiazoline-6-sulphonate (ABTS) radical. Trolox was used as a standard reference. The ABTS radical scavenging assay was determined following the method of Re et al. [43], with some modifications; the blue–green ABTS radical cation chromophore (ABTS **●^+^**) was prepared by reacting ABTS stock solution (7 mmol) with potassium persulphate (2.45 mmol) and allowing the mixture to stand at room temperature in the dark during 16 h. The solution was diluted with ethanol to obtain an absorbance of 0.700 ± 0.02 at 734 nm in a Genesys 5 (spectronic) spectrophotometer. 

The antioxidant activity was calculated as a percentage of inhibition according to the following Equation (2):

% Inhibition = {[Ab − As/Ab] × 100}(2)

Ab represents the absorbance of the control (without test oil), and as represents the absorbance of the test oil. A calibration curve was determined for Trolox standard for the radical (ABTS) at a concentration range of 10–160 μmol. The antioxidant activity values were expressed as μmol Trolox equivalent (TE)/mg oil.

#### 3.3.2. Antifungal Assay

The antifungal assay was performed according to the semi-quantitative method reported by Rosas-Taraco et al. [44] and Sánchez et al. [45] with some modifications. The strains of *Aspergillus flavus* and *A. niger* kindly provided by Dr. Eduardo Sánchez from Chemistry Laboratory of UANL, were used to determine the Fungal Inhibitory Concentration (FIC) and were maintained on potato dextrose agar (PDA) slants (Difco). Inoculum was prepared from PDA cultures incubated for seven days at 28 °C until they sporulated. Fungal conidia were collected with a loop and suspended in saline solution containing 0.05% of Tween 20 and adjusted to (1 × 10^4^) by microscopy in a Neubauer chamber. An amount of 200 μL of this suspension was added into tubes containing 1.8 mL of PDA broth plus inclusion complexes in different concentrations, ranging from 1.25 to 10 mg/mL. The tubes were incubated at 28 °C for 5 days. The antifungal activity was assigned according to a symbolic scale (+) and arbitrary from 0 to 3, where (-) represents total absence of growth, (+) represents little growth, (++) represents medium growth and (+++) represents high growth compared to the control growth of the pure strain without inclusion complexes. The FIC was defined as the minimum concentration that did not allow the visual mycelial growth of the fungus. Analyses with not encapsulated COEO were carried out to compare the activity before and after encapsulation. β–CD without COEO was used as negative control.

#### 3.3.3. Antibacterial Activity

Antibacterial activity of inclusion complexes was tested against *Salmonella typhi* (ATCC 19430) and *Listeria monocytogenes* (ATCC 13061), which were kindly provided by the Laboratory of Sanitary Microbiology, FCB, UANL. These strains were selected for their importance to the food industry and are representatives of pathogenic bacteria, commonly occurring in various food products. The strains were stored at −80 °C in brain heart infusion (BHI) with 20% *v/v* glycerol (Difco Laboratories, Sparks, MD, USA). Fresh cultures (18 h) were obtained in Mueller–Hinton broth or agar (MH, Difco Laboratories). *Listeria monocytogenes* was cultivated on trypticase soy broth or agar (TS, Difco Laboratories). The antimicrobial activity was determined through a dilution method according to Castillo et al. [46]. The minimum inhibitory concentration (MIC) was defined as the lowest concentration of inclusion complex that decreased growth by about 90% of the bacterial population after 24 h of incubation [34,47]. Aliquots (10 μL) of fresh and adjusted cultures (0.1 OD 600nm) (1 × 10^6^) of each bacterium were added separately in tubes containing 2 mL of MH broth plus different concentrations (1.25, 2.5, 5, and 10 mg/mL) of the inclusion complexes (5x, 10x, and 20x); after that, they were incubated at 37 °C for 24 h. Subsequently, plate counts were made using the Miles and Misra method. For *Listeria monocytogenes*, the same procedure was used, but TS medium instead of MH was used. The control was the same preparation without inclusion complexes. β–CD was used as a negative control while previous results of MIC of free COEO were used as a positive control [11].

### 3.4. Statistical Analysis

All experiments were analyzed in triplicate. Statistical analyses were performed using SPSS software (IBM version 22, SPSS Inc, Chicago, IL, USA). The results were expressed as means ± Standard Deviation (SD). Data were analyzed by analyses of variance test (one-way ANOVA) and a post hoc test of Tukey’s multiple range. Differences between means were considered significant at *p* values ≤ 0.05.

## 4. Conclusions

The obtention of inclusion complexes of concentrated orange oils (5x, 10x, 20x) was achieved by using the co-precipitation method. The results of this study suggest that concentrated orange oils were complexed with β–CD to form an inclusion complex in various ratios of 4:96, 12:88 and 16:84 (COEO: β–CD). The best recovery and entrapment efficiency (%) were found in the 12:88 ratio with 20x COEO. Moreover, FT-IR showed changes in spectra between free oil and inclusion complex (COEO: β–CD). The minimum inhibitory concentration was determined for bacteria at 1.25 mg/mL while for fungi it was higher (10 mg/mL). On the other hand, the antioxidant activity was lower than free oil but was detected in all ratios. This research provides information regarding the alternative use of these oils in the food industry. Future studies should be directed towards the determination of encapsulated compounds in β–CD and the confirmation of complex formation.

## Figures and Tables

**Table 1:** Recovery of the powder (complex) from each concentrated orange oil (COEO) to β-cyclodextrin (β–CD) ratio.

COEO: β–CD Ratio	Recovery (%) *		
	5x	10x	20x
4:96	78.4 ± 0.7 ^b,c^	79.00 ± 1.9 ^b,c^	74.4 ± 2.2 ^c^
12:88	83.3 ± 1.1 ^a,b^	81.2 ± 2.3 ^a,b^	80.9 ± 1.7 ^a,b^
16:84	84.5 ± 2.2 ^a^	84.8 ± 1.1 ^a^	82.0 ± 2.1 ^a,b^

* Values given are averages of three replicates ± standard deviations. Data were analyzed by an analysis of variance test (one-way ANOVA) and a post hoc test of Tukey’s multiple range. Means within a column which are not followed by a common superscript letter (a, b, c) are significantly different (*p* < 0.05).

**Table 2:** Entrapment efficiency (%) and encapsulated oil values (mg/g β–CD) for each COEO:β–CD ratio.

COEO	Ratio	Encapsulated Oil * (mg/g β–CD)	Entrapment Efficiency (%)
5x	4:96	7.8 ± 1.3 ^f^	75.6 ± 0.1 ^c^
	12:88	23.7 ± 1.6 ^e^	84.4 ± 3.0 ^a,b^
	16:84	35.8 ± 4.1 ^d^	85.2 ± 2.4 ^ab^
10x	4:96	7.7 ± 1.3 ^f^	84.5 ± 2.0 ^ab^
	12:88	58.1 ± 1.0 ^c^	80.7 ± 3.4 ^b,c^
	16:84	35.9 ± 2.2 ^d^	67.1 ± 3.4 ^d^
20x	4:96	18.2 ± 2.5 ^e^	85.8 ± 0.9 ^a,b^
	12:88	102.3 ± 3.4 ^a^	89.5 ± 0.4 ^a^
	16:84	83.1 ± 1.5 ^b^	74.5 ± 1.4 ^c^

* Values given are averages of three replicates ± standard deviations. Data were analyzed by an analysis of variance test (one-way ANOVA) and a post hoc test of Tukey’s multiple range. Means within a column which are not followed by a common superscript letter (a, b, c, d, e, f) are significantly different (*p* < 0.05) for each oil.

**Table 3:** Particle size (μm) values for each COEO: β–CD ratio.

COEO: β–CD Ratio	Particle Size (μm) *		
	5x	10x	20x
4:96	3.59 ± 0.03 ^a^	2.11 ± 0.02 ^a^	3.64 ± 0.7 ^a^
12:88	2.46 ± 0.01 ^b^	1.62 ± 0.01 ^c^	1.51 ± 0.01 ^c^
16:84	1.97 ± 0.01 ^c^	1.99 ± 0.07 ^b^	1.90 ± 0.01 ^b^

* Values given are averages of three replicates ± standard deviations. Data were analyzed by an analysis of variance test (one-way ANOVA) and a post hoc test of Tukey’s multiple range. ^a–c^ Means within a column which are not followed by a common superscript letter are significantly different (*p* < 0.05)

**Table 4:** Measure of mycelial growth in the presence of inclusion complexes (COEO: β–CD).

Fungus	Ratio	COEO	mg/mL				Control
			1.25	2.5	5	10	
*Aspergillus niger*	12:88	5x	+++	+++	+++	++	+++
		10x	+++	++	++	+	+++
		20x	+++	+++	+++	+++	+++
	16:84	5x	+++	++	++	++	+++
		10x	+++	++	+	+	+++
		20x	+++	+++	+++	+++	+++
	not encapsulated	5x	+++	+++	++	-	+++
		10x	++	++	+	-	+++
		20x	+++	+++	+	-	+++
*Aspergillus flavus*	12:88	5x	+++	+++	+++	++	+++
		10x	+++	+++	+++	++	+++
		20x	+++	+++	+++	+++	+++
	16:84	5x	+++	+++	+++	++	+++
		10x	+++	+++	+++	++	+++
		20x	+++	+++	+++	++	+++
	not encapsulated	5x	+++	+++	++	-	+++
		10x	+++	+++	+	+	+++
		20x	+++	+++	++	-	+++

High mycelial growth (+++); medium mycelial growth (++); little mycelial growth (+); abssence of mycelial growth (-).
